# Insights From the *Lactobacillus johnsonii* Genome Suggest the Production of Metabolites With Antibiofilm Activity Against the Pathobiont *Candida albicans*

**DOI:** 10.3389/fmicb.2022.853762

**Published:** 2022-03-07

**Authors:** Roberto Vazquez-Munoz, Angela Thompson, Jordan T. Russell, Takanori Sobue, Yanjiao Zhou, Anna Dongari-Bagtzoglou

**Affiliations:** ^1^Department of Periodontology, University of Connecticut Health Center, Farmington, CT, United States; ^2^Department of Psychiatry/Medicine, University of Connecticut Health Center, Farmington, CT, United States

**Keywords:** *L. johnsonii*, *Candida abicans*, biofilm, *Lactobacillus*, bacteriocin, anticandida

## Abstract

*Lactobacillus johnsonii* is a probiotic bacterial species with broad antimicrobial properties; however, its antimicrobial activities against the pathobiont *Candida albicans* are underexplored. The aim of this study was to study the interactions of *L. johnsonii* with *C. albicans* and explore mechanisms of bacterial anti-fungal activities based on bacterial genomic characterization coupled with experimental data. We isolated an *L. johnsonii* strain (MT4) from the oral cavity of mice and characterized its effect on *C. albicans* growth in the planktonic and biofilm states. We also identified key genetic and phenotypic traits that may be associated with a growth inhibitory activity exhibited against *C. albicans*. We found that *L. johnsonii* MT4 displays pH-dependent and pH-independent antagonistic interactions against *C. albicans*, resulting in inhibition of *C. albicans* planktonic growth and biofilm formation. This antagonism is influenced by nutrient availability and the production of soluble metabolites with anticandidal activity.

## Introduction

*Candida albicans* is the most common opportunistic fungal pathogen in immunocompromised hosts (Pfaller and Diekema, 2010; Pfaller et al., 2019). Although it is a member of the mucosal microbiota at different body sites in health, under certain conditions, it can cause invasive mucosal infections (Pendleton et al., 2018). The ability of *C. albicans* to switch from yeast to hyphal morphotypes and form biofilms increases its virulence on mucosal tissues (Pappas et al., 2018). The biofilm growth form also increases resistance to innate immune effector cells and antimicrobial treatments (Dongari-Bagtzoglou et al., 2009; Vazquez-Munoz et al., 2020).

The clinical outcome of mucosal candidiasis has been adversely affected by the rise of drug-resistant *C. albicans* strains which have become a serious threat to human health (Centers for Disease Control and Prevention, 2019). As the effectiveness of conventional antifungals is diminished, novel strategies are being developed, such as probiotic therapies (Mundula et al., 2019). In this regard, several species from the *Lactobacillus* complex genus have been studied as probiotic therapies in gastrointestinal, vulvovaginal, and oral *Candida* infections in mouse models and human clinical trials (Vazquez-Munoz and Dongari-Bagtzoglou, 2021). This is due to the fact that certain lactobacilli produce soluble metabolites—i.e., bacteriocins, weak organic acids (such as lactic and acetic acids), and biosurfactants with anticandidal properties (Vazquez-Munoz and Dongari-Bagtzoglou, 2021). Antifungal activities vary across *Lactobacillus* species and even among strains (Strus et al., 2005; Jang et al., 2019); hence, the studies on relatively unexplored species, such as *Lactobacillus johnsonii*, and newly isolated strains within this species are novel and can unravel potentially significant probiotic properties.

Recently our group reported that the virulence of *C. albicans* in a mouse model of oropharyngeal candidiasis is attenuated by dietary sucrose. Sucrose significantly enriched the *L. johnsonii* communities on the oral mucosa during infection with *C. albicans* and caused a reduction in fungal burdens (Bertolini et al., 2021). Since other *Lactobacillus* species are antagonistic to *Candida* (Vazquez-Munoz and Dongari-Bagtzoglou, 2021), we hypothesized that *L. johnsonii* might exert a growth inhibitory effect on *C. albicans*. *Lactobacillus johnsonii* are Gram-positive, facultatively anaerobic, non-motile bacteria that are part of the *L. acidophilus* group. Like other lactobacilli, *L. johnsonii* is considered as a GRAS (generally recognized as safe) microorganism and is regarded as a probiotic (Marcial et al., 2017; Zheng et al., 2020). *Lactobacillus johnsonii* is a member of the human health-associated gastrointestinal and vaginal mucosal microbiota, two sites afflicted by mucosal candidiasis (Fujisawa et al., 1992; Assefa et al., 2015; Zheng et al., 2020). However, there is scant information on the interactions between *L. johnsonii* and *C. albicans*, and the limited available information is contradictory (Gil et al., 2010; Assefa et al., 2015; Eryilmaz et al., 2019).

We recently isolated *L. johnsonii* strain MT4 from the oral mucosa of mice receiving a sucrose-enriched diet. This strain was identified as *L. johnsonii via* whole 16S rRNA gene sequencing (Bertolini et al., 2021). Human clinical trials suggest that *L. johnsonii*-based probiotics may reduce the burden of certain infections and metabolic disorders (Cruchet et al., 2003). In this work, our aim was to functionally characterize the genome of strain MT4, assess its effects on the planktonic and biofilm growth of the pathobiont *C. albicans*, and explore relevant mechanisms of antifungal activity. We discovered that *L. johnsonii* MT4 displays pH-dependent and pH-independent anticandidal properties, mediated by the release of soluble metabolites, resulting in inhibition of *C. albicans* planktonic growth and biofilm formation. Our results also shed new light on existing contradictory data regarding the impact of lactic acid-mediated acidification on *C. albicans* growth.

## Materials and Methods

### Strains

*Lactobacillus johnsonii* strain MT4 was reactivated from frozen stocks in 5 ml MRS broth (Difco™) in an anaerobic chamber at 37°C overnight. *Candida albicans* SC5314 (ATCC MYA-2876), also reactivated from frozen stocks, was sub-cultured in YPD broth (Yeast Extract, Sigma®; Bacto™ Peptone, Gibco; and Dextrose, J.T.Baker®), and incubated aerobically at 30°C in an orbital shaker, overnight. Bacterial and fungal overnight cultures were washed in PBS and adjusted to their final concentration in either MRS, MRS w/o dextrose (USbiological), BHI (BBL™, BD), or biofilm medium (RPMI medium 1640 [Gibco] supplemented with 10% BHI, and 10% Fetal Bovine Serum [Gibco]), as described below. In some experiments, *L. johnsonii* type strain ATCC 33200 was used for comparison.

### Genomic Characterization of *Lactobacillus johnsonii* MT4 Strain

Genome sequencing, taxonomy, and comparative genomics of *L. johnsonii* MT4 strain were assessed as follows: (I) *Sequencing*: DNA was extracted using the DNeasy® Blood & Tissue Kit (Qiagen, United States; Bertolini et al., 2021). The extracted genomic DNA was assessed for concentration and size using the Qubit 3.0 HS dsDNA assay (Life Technologies, Carlsbad, CA, United States) and the Tapestation 4200 genomic DNA assay (Agilent Technologies, Santa Clara, CA, United States), respectively. The DNA sample was diluted to 0.2 ng/μl, and the sequencing library was prepared using the Illumina Nextera XT DNA kit (Illumina, San Diego, CA, United States) according to the manufacturer’s instructions. The library was again checked for concentration and size (450 bp average library length; average insert size of 315 bp) as before, and the sequencing library was prepared using the Illumina Nextera XT DNA kit (Illumina, San Diego, CA, United States) and sequenced using 2 × 150 bp format on an Illumina NovaSeq 6000 at the Center for Genome Innovation (Institute for Systems Genomics, University of Connecticut). Reads pertaining to 1% PhiX control spike-in were filtered and removed *via* USEARCH v11.0.667. The Whole Genome Sequence (WGS) of MT4 was assembled using Unicycler v0.4.8, which utilizes the SPAdes assembler v3.15.2 (Bankevich et al., 2012), at the University of Connecticut’s Xanadu High-Performance Computing Cluster; (II) *Taxonomy*: the contigs resulting from the assembly of MT4 were taxonomically classified using Kraken2 v2.0.8-beta (Wood et al., 2019). Phylogenetic analysis on core genes was performed to find the closest *L. johnsonii* MT4 strain relatives. Seventeen whole-Genome Sequences of *L. johnsonii* strains from the NCBI database (strains NCK2677, ATCC 33200, N6.2, 3DG, BS15, Byun_jo_01, DC22.2, DPC_6026, FI9785, G2A, GHZ10a, IDCC9203, NCC_533, UMNLJ21, UMNLJ22, ZLJ010, and pf01) were compared. The assembled MT4 genome and the NCBI genomes were annotated with Prokka v1.14.6 (Seemann, 2014), and the comparative genomics analysis was performed with Roary v3.13.0 (Page et al., 2015) to identify genes unique and in common between our MT4 isolate and the other 17 NCBI genomes of *L. johnsonii* strains. Roary generates an alignment of the core genes using PRANK (Löytynoja, 2014). Phylogenetic analysis of the *L. johnsonii* strains was done using SeaView v5.0.4 (Gouy et al., 2010). The core gene alignment was curated for further analysis using GBlocks (Castresana, 2000) to remove poorly aligned regions, including large gaps. The phylogeny was generated with PhyML (Guindon et al., 2010) using the GTR DNA substitution model, which was determined as the optimal model using the Smart Model Selection (SMS) tool web server (Lefort et al., 2017) and branch support values calculated using the aLRT method.^1^ Nucleotide equilibrium frequencies, invariable sites, and across-site rate variation were set to “optimized,” with the BioNJ option selected (Gascuel, 1997). Finally, SeaView uses the PHYLIP package (Felsenstein, 1993) for tree parsimony. The presence or absence of genes and proteins in MT4 was also verified with searches using BLAST (Altschul et al., 1990) and tblastn, respectively.

### Phenotypic Characterization of *Lactobacillus johnsonii*

#### Growth on Different Culture Media

*Lactobacillus johnsonii* was grown on MRS (BD), BHI (BD), YPD, KSFM (Gibco), and F-12 (Gibco). Lactobacilli were incubated static, aerobically with 5% CO_2_, at 37°C. Optical density (*λ* = 600 nm) was measured every 90 min. The pH of growth media was measured at *t* = 24 h.

#### Aggregation

*Lactobacillus johnsonii* MT4 auto-aggregation phenotype was assessed in MRS broth after growth under aerobic conditions with 5% CO_2_, at 37°C, for 24 h. Auto-aggregation phenotype was defined as positive if the overnight cultures settled at the bottom with no turbidity and negative if they showed turbidity (Jankovic et al., 2003). The non-aggregating *L. johnsonii* ATCC 33200 strain was used as a negative control.

#### Assessment of D/L-Lactate Production

The DL-lactate kit (Megazyme) was used following the manufacturer’s recommendations with minor changes. Briefly, supernatants from overnight *L. johnsonii* and *C. albicans* in single-or dual-species cultures were deproteinized with ice-cold 1 M hydrochloric acid 1 M NaOH, and 20 μl of each sample was tested in duplicate. Standard curves were prepared in the corresponding culture medium. Absorbance was measured at *λ* = 340 nm, and D−/L-lactate concentrations were calculated in two independent experiments.

### Effect of *Lactobacillus johnsonii* on *Candida albicans* Planktonic Growth

*C. albicans* (5 × 10^4^ cells ml^−1^) and *L. johnsonii* (5 × 10^4^ to 5 × 10^8^ cells ml^−1^) were cocultured in MRS or BHI broth aerobically with 5% CO_2_ for 24 h, at 37°C. These growth conditions allow planktonic growth of *C. albicans* exclusively in the yeast form. The influence of media acidification on *C. albicans* growth was assessed in lactic acid-(DL-LA, Sigma-Aldrich)-supplemented MRS broth (pH 4.0 ± 0.05, 142 mM) or BHI broth (pH 5.5 ± 0.08, 28.3 mM). To assess the influence of carbohydrate availability, single- or dual-species cultures were tested in MRS broth without dextrose (MRSm) or with added carbohydrates (2% sucrose or 2% dextrose). At the end of each culture period, *L. johnsonii* viable counts (CFU) were estimated by plating serially 10-fold diluted cultures onto MRS agar plates, incubated anaerobically at 37°C, for 48 h. *C. albicans* yeast cell numbers were assessed by counting in a Neubauer chamber after fixation in 1% paraformaldehyde (PFA, Sigma).

### Preparation of Non-viable *Lactobacillus johnsonii*

Some studies have reported that exopolysaccharides from the cell wall of UV-inactivated bacterial cells may decrease the burden of *Candida* infections (Wagner et al., 2000) and reduce the dimorphic transition (Allonsius et al., 2019). Thus, the impact of inactivated *L. johnsonii* cells on *C. albicans* was evaluated. *Lactobacillus johnsonii* cells were UV-light-inactivated in a UVP crosslinker instrument (Analytik Jena; 254 nm, 1,500 mJ cm^−2^, at 8 cm from the UV lamp) for 12 cycles; Heat-killed organisms were prepared at 95°C for 30 min on a heat block (VWR). To verify that bacteria were killed, 10 μl from each suspension were transferred into 1 ml of MRS and incubated at 37°C for 48 h. *Candida albicans* (~5 × 10^4^ cells ml^−1^) with killed lactobacilli (~5 × 10^6^ cells ml^−1^) were suspended in BHI broth and were incubated as described above.

### Preparation of Cell-Free Supernatants

Supernatants from *L. johnsonii* single-species cultures (Lj-cell-free supernatant (CFS), starting at 5 × 10^7^ cell ml^−1^) or in cocultures with *Candida albicans* (CC-CFS, starting at 5 × 10^4^ cell ml^−1^) were prepared in MRS broth, BHI broth, or biofilm medium under aerobic conditions with 5% CO_2_, at 37°C, for 24 h. CFS were collected by centrifugation at 4,000 rpm for 20 min. The pH of supernatants was measured, and each supernatant was divided into two aliquots; one was kept at the original pH (acidic-CFS, pH 3.87 ± 0.03 for MRS broth, 5.40 ± 0.04 for BH, and pH 6.08 ± 0.18 for biofilm medium), while the other was pH-adjusted with a 1 M NaOH solution (neutralized-CFS, pH 6.6 ± 0.14 for MRS, pH 7.4 ± 0.06 for BHI, and pH 8.68 ± 0.04 for biofilm medium). CFS were sterile filtered using a 0.2 μm filter (Corning). CFS were stored at 4°C. The effect of CFS on *C. albicans* (~5×10^4^ cells ml^−1^) was tested in growth media supplemented with 50% CFS or PBS as control.

### Impact of *Lactobacillus johnsonii* on *Candida albicans* Biofilm Growth

#### Biofilm Growth

*Lactobacillus johnsonii* (5 × 10^5^ to 5 × 10^7^ cells ml^−1^) and *C. albicans* (5×10^4^ cells ml^−1^) were suspended in biofilm medium (80% RPMI, 10% BHI, and 10% FBS) and seeded in multiwell plates or into μ-Slide 8-well chambered slides (IBIDI GmbH, Gräfelfing, Germany) and incubated statically, aerobically with 5% CO_2_ at 37°C for up to 48 h. Single-species cultures were used as control. To visualize *L. johnsonii*, bacteria were stained with 1 mM CellTracker™ Deep Red dye (Thermo Fisher Scientific). Fungal cells were stained with Calcofluor White for 10 min, washed in PBS, and fixed with 4% paraformaldehyde (PFA). The impact of physical contact between the lactobacilli and *Candida* on fungal biofilm growth was assessed by seeding *L. johnsonii* (5 × 10^6^ cell ml^−1^) into a Millicell® 0.4 μm PCF Cell Culture Insert (Millipore, United States). The inserts were placed into the wells of 24-well plates (Corning) containing *C. albicans* (5 × 10^4^ cell ml^−1^). Biofilms were grown for 24 h, as above. Exclusion and displacement experiments were performed to assess the effect of preformed biofilms on the ability of the other microbial species to form a biofilm. The supernatant was removed, and preformed biofilms were washed twice with PBS. In exclusion assays, *C. albicans* (5 × 10^4^ cells ml^−1^) suspended in fresh biofilm medium was transferred to preformed *L. johnsonii* biofilms. In displacement assays, *L. johnsonii* (5 × 10^7^ cells ml^−1^) suspended in fresh medium was added to preformed *C. albicans* biofilms. For negative controls, cell-free medium was added to the single-species 24 h-old preformed biofilms. Plates were incubated for an additional 24 h, as described above.

#### Biofilm Analyses

##### Biovolumes and Thickness

Micrographs were obtained at a ×400 magnification in a Microscope (Zeiss), with the DAPI (*λ*_exc_ = 358 nm, *λ*_em_ = 463 nm) and CY3 (*λ*_exc_ = 549 nm, *λ*_em_ = 562 nm) fluorescence channels and using the Z-stack mode. Images were post-processed in Zen Blue (v.3.0, Zeiss) and analyzed with IMARIS Cell Imaging Software (Oxford Instruments) using the Create Surface tool to reconstruct 3D images from the biofilms and assess their volumes.

##### Biomass

Fungal biomass was quantified by qPCR. DNA from biofilms grown on 24-multiwell plates was extracted using the Yeast DNA Extraction Kit (Thermo Scientific), following the manufacturer’s recommendations. A region from the fungal rRNA operon was amplified using the primers 5.8S GTGAATCATCGARTCTTTGAAC (forward primer) and 28S-1 TATGCTTAAGTTCAGCGGGTA (reverse primer) under the qPCR conditions reported by Khot et al. (2009). Fungal biomass was directly correlated to the number of amplicons. Each experiment contained untreated biofilms and cell-free media as controls.

##### Metabolic Activity

The fungal metabolic activity was assessed *via* XTT as described elsewhere (Pierce et al., 2008). Briefly, biofilms were incubated for 30 min with Penicillin G/Streptomycin (100 μg ml^−1^, Gibco) to remove the metabolic signal from lactobacilli. Then, biofilms were washed with BPS, XTT/menadione was added, and samples were incubated for 2.5 h. Absorbance was read at 490 nm.

#### Reproducibility and Statistical Analyses

Data from at least two independent experiments with technical replicates were analyzed for statistical significance using One-Way ANOVA with Kruskal-Wallis posttest in Prism v9.2.0 (GraphPad Software, LLC).

## Results

### Genomic Characterization of *Lactobacillus johnsonii* Strain MT4

**Figure 1:**
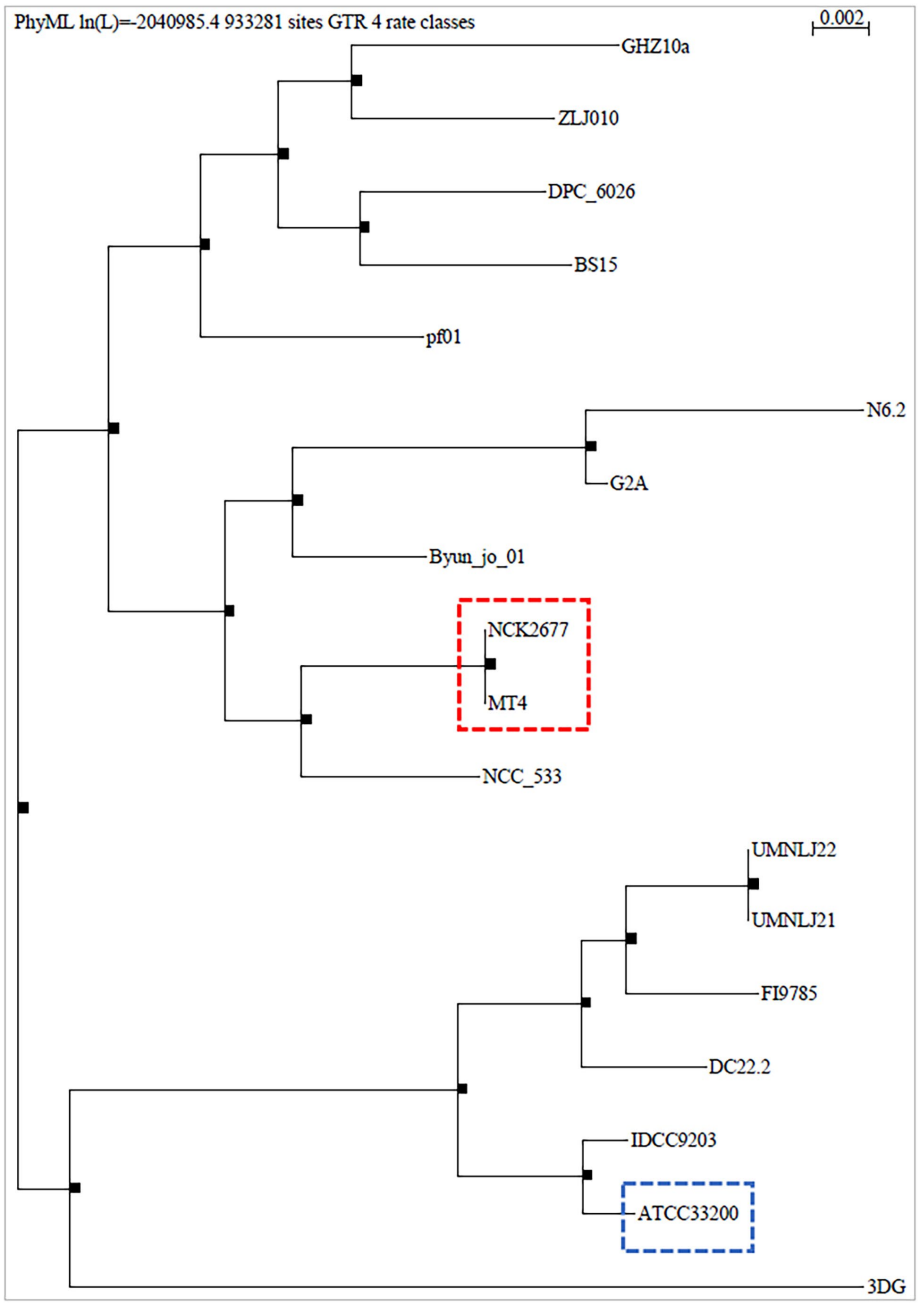
Phylogenetic analysis of *Lactobacillus johnsonii* MT4. The whole genome of the *L. johnsonii* MT4 strain was analyzed to assess its phylogeny. The phylogenetic analysis of the MT4 strain shows the relation of our strains with other 17 *L. johnsonii* strains (genomes from the NCBI database). The analyzed *L. johnsonii* strains are NCK2677, ATCC 33200, N6.2, 3DG, BS15, Byun_jo_01, DC22.2, DPC_6026, FI9785, G2A, GHZ10a, IDCC9203, NCC_533, UMNLJ21, UMNLJ22, ZLJ010, and pf01. All branch support values are 1. The phylogenetic analysis shows that MT4 and NCK2677 share >99.96% of identity (red square). The closest relative to strain MT4/NCK2677 is NCC_533 (La1). ATCC 33200 strain (blue square) was used for some experiments as a comparison.

The MT4 genome assembly resulted in 68 contigs with an N50 of 90.96 kb. All but 1 contig were classified as *L. johnsonii*, with a single contig classified as *L. crispatus* based on the Kraken2 results (Supplementary Table 1). The 1,883,026 bp genome has a GC content of 34.4%. In total, 1,865 genes were predicted, including 1,772 protein-coding genes and 93 RNA genes (34 miscellaneous/non-coding RNA, 3 rRNA, 55 tRNA, and one tmRNA). Phylogenetic analysis on core genes shows the relation of this strain to 17 *Lactobacillus johnsonii* strains deposited in the NCBI database (Figure 1), indicating that strain MT4 is almost identical to strain NCK2677, also isolated from the GI tract of C57BL/6 mice (O’Flaherty et al., 2020), sharing more than 99.96% identity of their genomes. The closest relative to the MT4/NCK2677 strains is NCC 533 (La1), a strain with probiotic properties (Yamano et al., 2006). Functional genomics analysis revealed that strain MT4 possesses genes encoding products that are similar to reported anticandidal metabolites (a bacteriocin, two hydrolases, and a biosurfactant; Table 1). The alignment analysis is listed in Supplementary Table 2. MT4 also possesses genes for both D- and L-Lactate synthesis and other metabolites of interest, such as the bacteriocins lactacin-F and helveticin J, and a glucanase (glycoside hydrolase family 8).

**Table 1:** *Lactobacillus johnsonii* strain MT4 possesses genes encoding for metabolites similar to products reported to display anticandidal activity.

Metabolite	Type	Reported anticandidal effect	References
Bacillomycin D	Lipopeptide, Bacteriocin	Disruption of the fungal cell membrane by forming ion-conducting pores. Bacillomycin D-like peptides inhibit the β-1,3-glucan synthesis.	Olfa et al., 2015; Hajare et al., 2016
Surfactin	Cyclo-lipopeptide biosurfactant	Prevent fungal adhesion to surfaces, reducing biofilm formation	Nelson et al., 2020
Glucanase	Glycoside hydrolase family 8	Hydrolysis of O-glycosyl compounds from *Candida* cell wall.	Jung, 2018
Msp1/p75	Peptidoglycan hydrolase	Chitinase activity; degrades *Candida* cell wall and inhibits hyphal morphogenesis	Allonsius et al., 2019

### Phenotypic Traits of *Lactobacillus johnsonii* MT4

#### Growth on Different Media

*Lactobacillus johnsonii* MT4 displayed the best aerobic growth rate on MRS broth, followed by BHI broth, while it displayed poor growth in all other media (Supplementary Figure 1A).

#### Auto- and Co-aggregation

**Figure 2:**
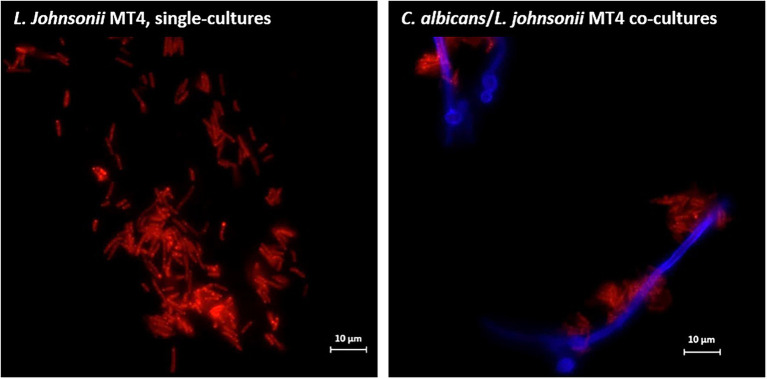
*Lactobacillus johnsonii* co-aggregates with *Candida albicans* cells. *L. johnsonii* (red) auto-aggregates and co-aggregates with *C. albicans* (blue). The yeast and the lactobacilli were grown on biofilm media for 3 h. Lactobacilli were found in physical proximity with *Candida* cells, particularly along the hyphae. Scale bar = 10 μm.

Overnight cultures of *L. johnsonii* strain MT4 showed a partial degree of auto-aggregation, whereas the strain ATCC 33200 did not (Supplementary Figure 1B). Additionally, *L. johnsonii* MT4 co-aggregated with *C. albicans* cells in dual-species biofilms, particularly on hyphae (Figure 2).

#### Acidification

**Figure 3:**
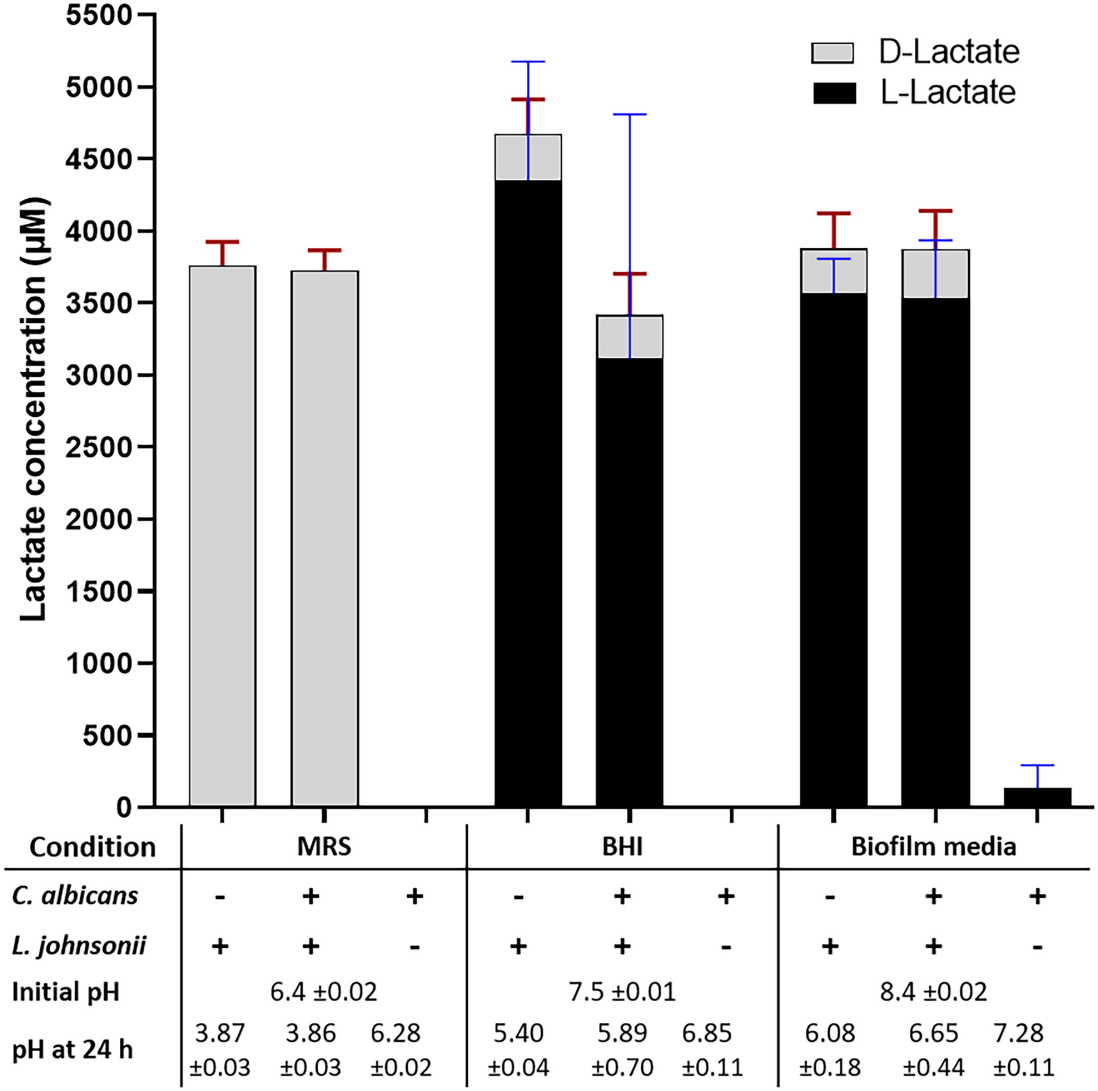
Lactate production and pH on different culture media. The production of DL-lactate by the MT4 strain was measured in various growth media. The growth media influence DL-Lactate production and ratio. In MRS, the D enantiomer is favored, while the L enantiomer production takes over in BHI and biofilm media. Acidification of the culture media does not correspond to the total DL-lactic acid concentration, which may be due to the particular buffering capacity of each growth media and the presence of other organic acids.

Growth of *L. johnsonii* MT4 in MRS, BHI, and biofilm medium (80% RPMI, 10% BHI, 10% FBS) displayed a different degree of acidification in overnight cultures (Figure 3, bottom table). Regardless of the starting inoculum size (10^4^–10^7^ cell ml^−1^ range), lactobacilli acidified MRS broth to pH 3.9 (from 6.5), BHI to pH 5.5 (from 7.5), and biofilm medium to pH 6 (from 8.4). In addition to having different starting pH, the difference in pH at the end of the growth period in the two media may be associated with a different buffering capacity or the production of different amounts of organic acids in each medium. *C. albicans* had a small neutralizing effect on the pH of the growth media in dual-species cultures with *L. johnsonii* in BHI and biofilm medium only (Figure 3).

#### DL-Lactate Production

*Lactobacillus johnsonii* is reported to produce both the D and L enantiomers of lactate (Fujisawa et al., 1992). The functional genome analysis and a DL-lactate assay confirmed that strain MT4 produces both enantiomers (Figure 3). Interestingly, the type of growth medium influenced the D−/L-lactate ratio; D-lactate was favored in MRS broth with both MT4 (Figure 3) and ATCC 33200 (Supplementary Figure 2A) strains, whereas L-lactate was predominant in BHI broth and biofilm medium, representing 93% and 92% of the total production, respectively. The total DL-lactate production was similar in all growth media, ranging between 3.5–4.5 mM (Figure 3). *Candida albicans* lactate production was negligible and did not influence the lactate production of *L. johnsonii* (Figure 3).

### *Lactobacillus johnsonii* Inhibits *Candida albicans* Planktonic Growth

**Figure 4:**
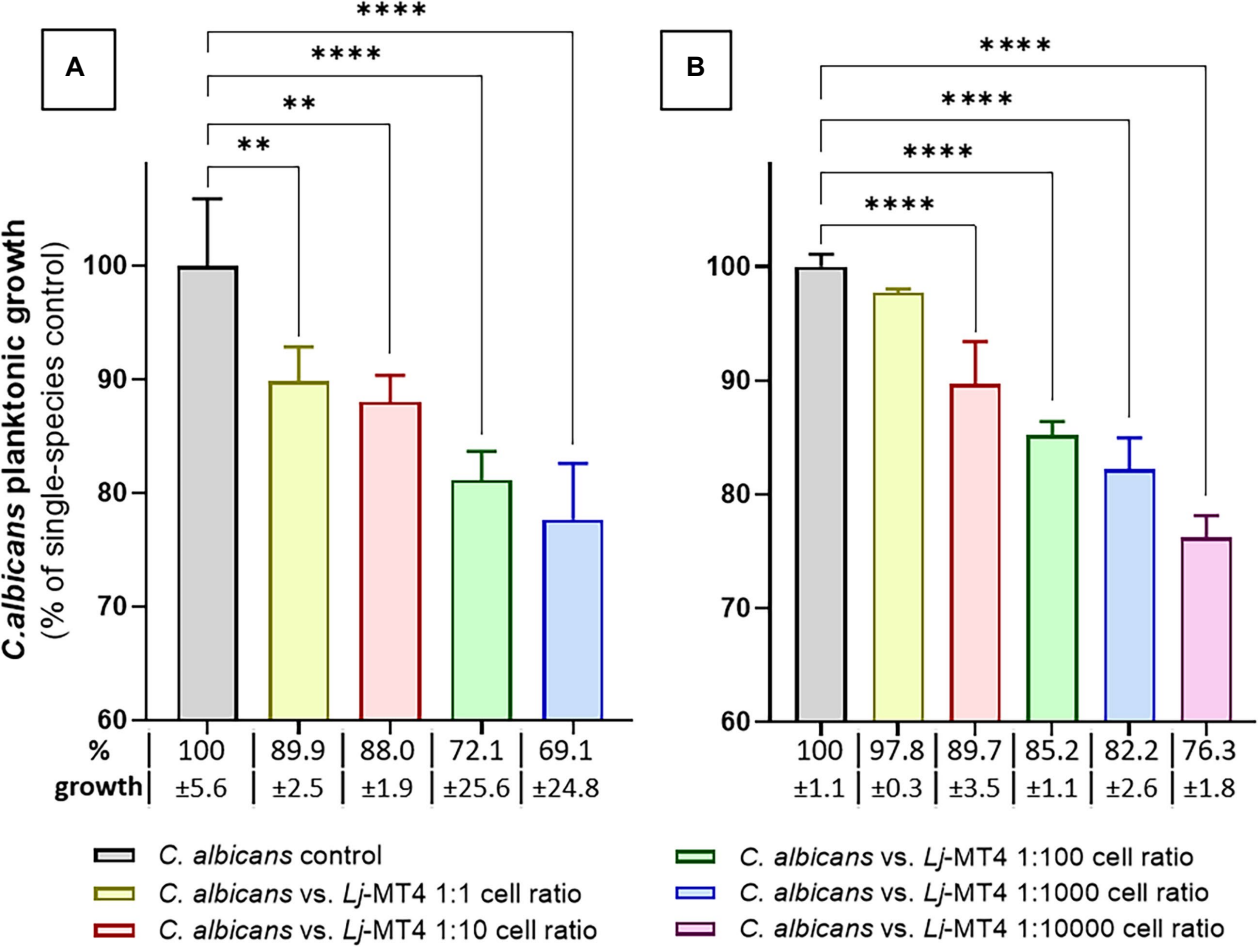
*Lactobacillus johnsonii* inhibits *C. albicans* planktonic growth. The effect of *L. johnsonii* MT4 (Lj) on the planktonic growth of *C. albicans* (Ca) was assessed in two different growth media. *L. johnsonii* at different starting concentrations-inhibited *C. albicans* planktonic growth in a dose–response pattern. **(A)** MRS broth, and **(B)** BHI broth. *Candida albicans* growth inhibition is higher on MRS. One-Way ANOVA, Dunnett posttest, ** *p* ≤ 0.01 and **** *p* ≤ 0.0001. In all dual-species cultures, MRS was acidified to pH ~3.9 and BHI to pH ~5.5. 100% growth corresponds to 8.16 ± 0.41 SD and 7.47 ± 0.08 SD yeast cells ml^−1^ (average, log10 values) in MRS and BHI broth, respectively.

We first tested the effect of *L. johnsonii* MT4 on *C. albicans* planktonic growth in the two media in which this strain showed the best growth. In both MRS and BHI broth *L. johnsonii* inhibited *C. albicans* growth following a dose-repose trend (Figures 4A,B). The inhibition of *C. albicans* growth was more pronounced in MRS, which also showed higher acidification at the end of the coculture period (MRS pH 3.9 vs. BHI pH 5.5). *Lactobacillus johnsonii* ATCC 33200 displayed similar anticandidal activity in MRS broth (Supplementary Figure 2B).

To assess the role of acidification in inhibiting fungal growth, we supplemented the media with sufficient amounts of lactic acid to lower the pH to levels comparable with a 24 h culture of *L. johnsonii*. *Candida albicans* growth inhibition in lactic acid-supplemented MRS broth was similar to *L. johnsonii*-induced inhibition, showing that lactic acid-induced acidic pH alone is sufficient to cause growth inhibition in this medium. In contrast, growth inhibition in lactic-acid supplemented BHI was significantly lower than that induced by live bacteria suggesting that acidic pH alone is not responsible for the growth inhibition observed in this medium (Supplementary Figure 3).

### Carbohydrate Availability Plays a Role in *Candida albicans* Growth Inhibition in MRS Broth

**Figure 5:**
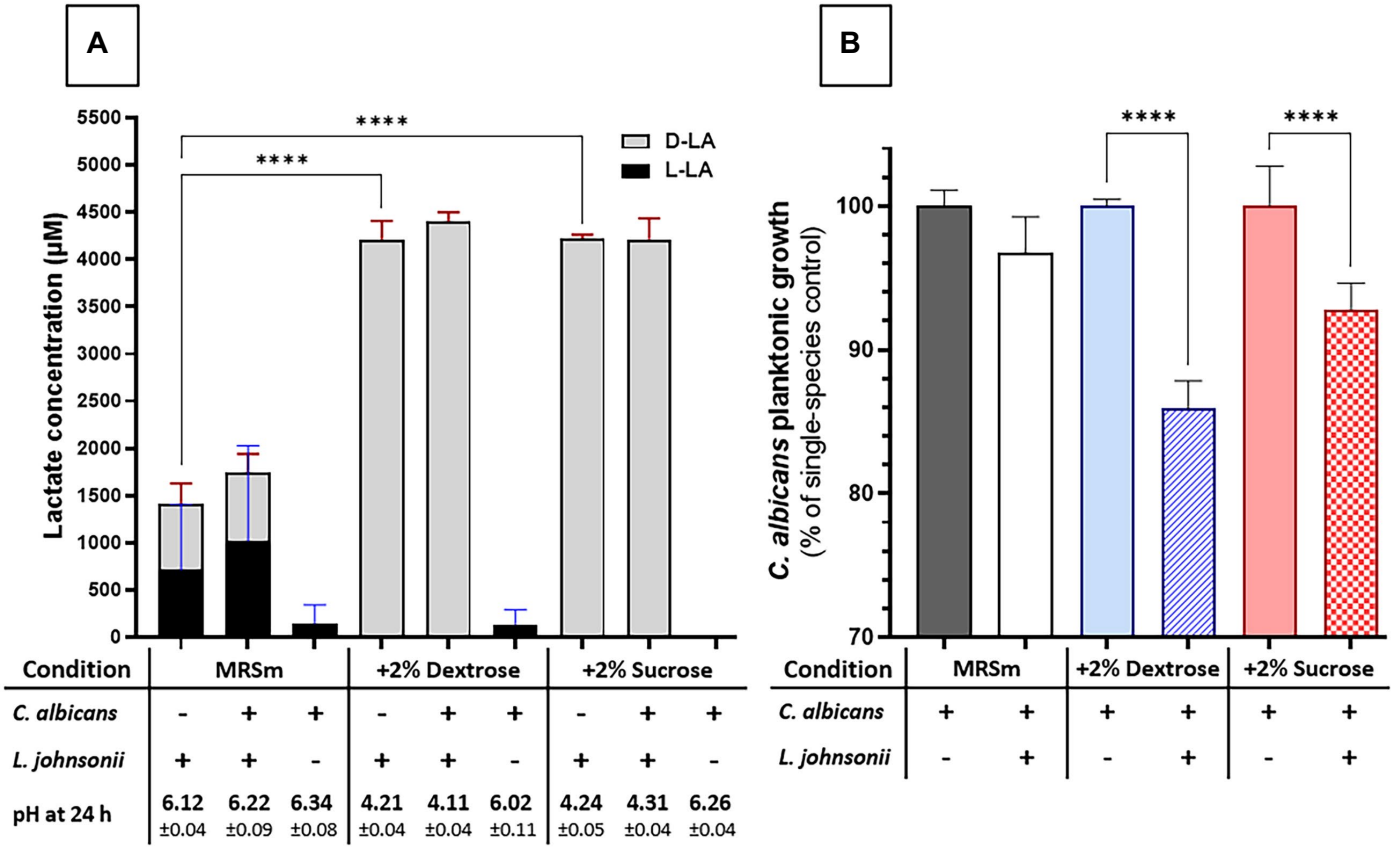
*C*arbon sources influence *L. johnsonii* anticandidal activity, lactic acid production, and pH. **(A)** Lactate production is impacted by availability but not by the type of carbohydrate (i.e., sucrose vs. glucose). The acidification of the culture media correlates to lactate production; **(B)** The carbohydrate availability and its type influenced the anticandidal activity of *L. johnsonii*. One-Way ANOVA, Dunnett posttest, **** *p* ≤ 0.0001. 100% growth corresponds to 6.51 ± 0.06 SD, 7.37 ± 0.02 SD, and 6.71 ± 0.06 SD yeast cells ml^−1^ (average, log10 values) in MRSm, MRSm+2% dextrose, and MRSm+2% sucrose, respectively.

**Figure 6:**
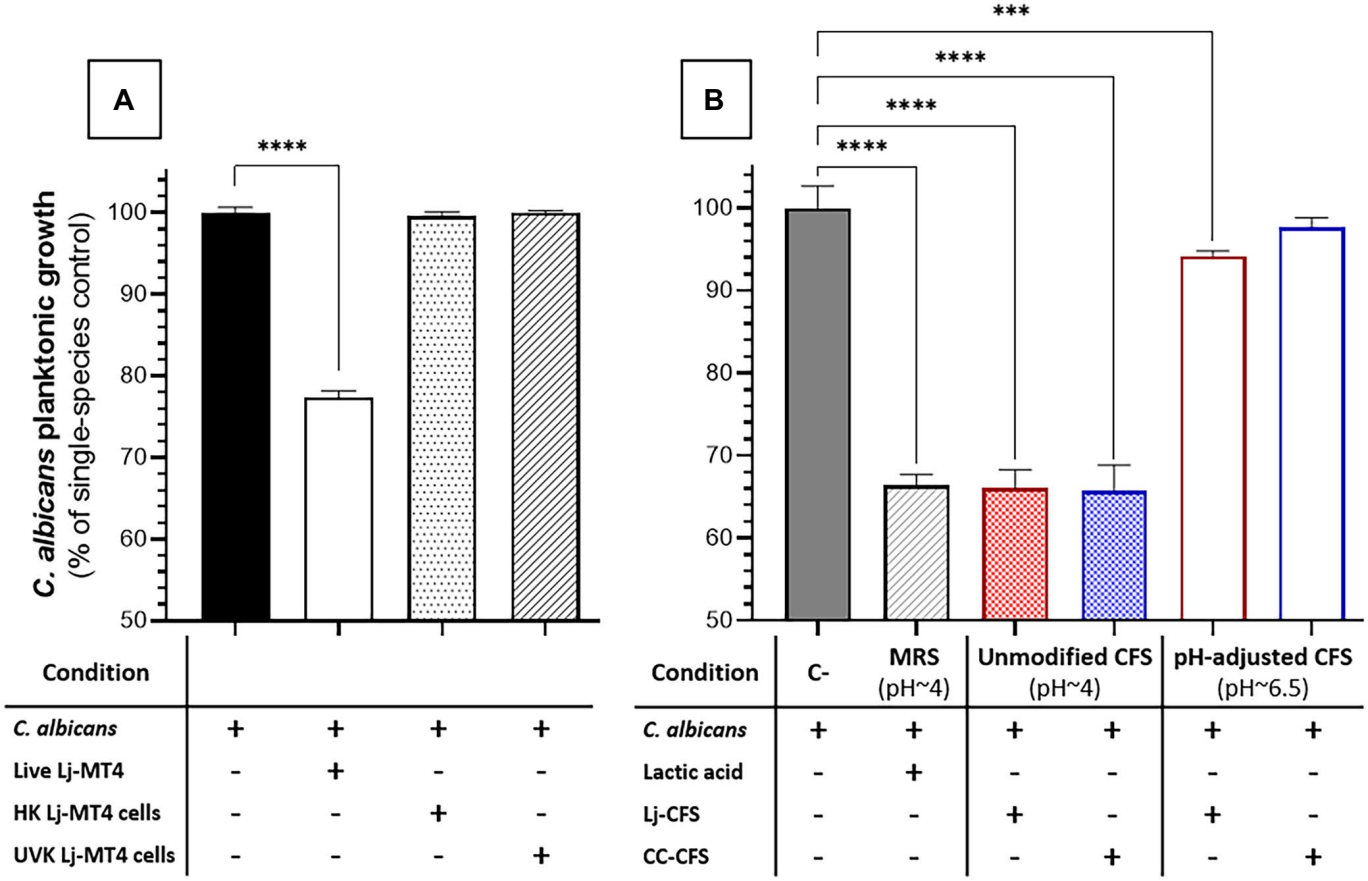
Effect of *L. johnsonii* inactivated cells and soluble metabolites on *C. albicans* growth in MRS broth. **(A)** Inactivated *L. johnsonii* cells did not reduce the growth of *C. albicans*; **(B)** *L. johnsonii* CFS inhibited the growth of *C. albicans* in a pH-dependent manner. HK = heat killed, UVK=UV-killed. CFS = Cell-free supernatant (spent media). One-Way ANOVA, Dunnett posttest, *** *p* ≤ 0.005, **** *p* ≤ 0.0001. 100% growth corresponds to 8.16 ± 0.41 SD yeast cells ml^−1^ in MRS broth.

We next evaluated the impact of carbohydrate availability on *Lactobacillus* fitness and its ability to inhibit *Candida* growth in MRS broth. In carbohydrate-free MRSm, there was a significantly reduced *L. johnsonii* growth rate (Supplementary Figure 4), lactate production was significantly curtailed, and the pH of the growth media remained close to the initial pH (Figure 5A). As expected, supplementing the media with either 2% dextrose or 2% sucrose fully restored lactate production and caused media acidification, while co-culture with *C. albicans* did not affect the amounts of lactate produced (Figure 5A). In MRS broth, growth inhibition of *C. albicans* by *L. johnsonii* MT4 required the availability of carbohydrates (Figure 5B), being higher in the 2% dextrose-supplemented MRSm compared to the non-supplemented or 2% sucrose-supplemented MRSm. Since the *Candida*-inhibitory effect of *L. johnsonii* was significantly curtailed when bacterial carbohydrate metabolism was suppressed in MRSm, we hypothesized that bacterial viability is also required for anticandidal activity. As expected, neither UV- nor heat-killed *L. johnsonii* inhibited *C. albicans* yeast growth in MRS broth (Figure 6A). Collectively these results indicate that carbohydrate availability influences the ability of *L. johnsonii* to produce weak organic acids and metabolites with anticandidal properties that may be responsible for the growth inhibition of the yeast in MRS.

To further dissect the role of soluble metabolites released in culture media on the growth inhibition observed in MRS broth, we tested the effect of CFS (i.e., *Lactobacillus* spent media) on *C. albicans* growth. CFS from *Lactobacillus* cultures in MRS broth showed significant anticandidal activity, similar to co-cultures with live lactobacilli or lactic acid-acidified media (Figures 6A,B). However, the pH-adjusted CFS (pH ~6.5) from *Lactobacillus* cultures in MRS broth had significantly reduced anticandidal activity, suggesting that acidic pH is required for most anticandidal activity of *Lactobacillus* metabolites in MRS broth. Similar results were obtained with CFS prepared from cocultures of *C. albicans* and *L. johnsonii* (Figure 6B).

### *Lactobacillus johnsonii* Reduces the Ability of *Candida albicans* to Form Biofilms

**Figure 7:**
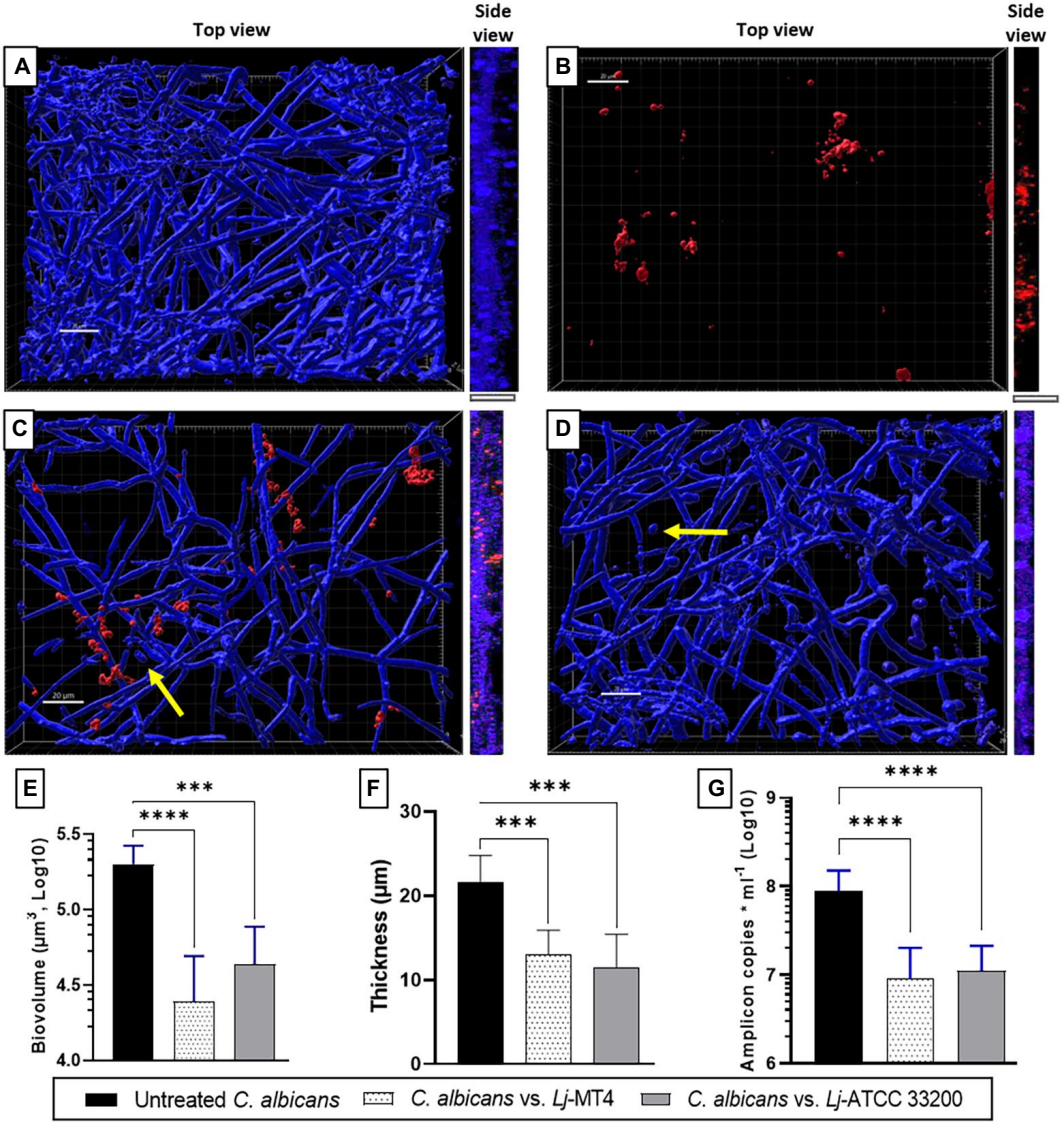
*Lactobacillus johnsonii* reduces the ability of *C. albicans* to form biofilms. **(A)** Single *C. albicans* biofilms uniformly cover the surface; **(B)** Single *L. johnsonii* biofilms consist of scattered clusters of cells; *C. albicans* biofilm formation is reduced by *L. johnsonii* **(C)** MT4 and **(D)** ATCC 33200. Fungal biofilm **(E)** biovolumes, **(F)** thickness, and **(G)** biomass are significantly reduced in the presence of lactobacilli. Yellow arrows indicate the presence of yeast cells. White bar = 20 μ. One-Way ANOVA, Dunnett posttest. *** *p* ≤ 0.005, **** *p* ≤ 0.0001.

Single species *C. albicans* biofilms uniformly covered the well surfaces (Figure 7A), while *L. johnsonii* MT4 biofilms consisted of scattered clusters of cells (Figure 7B). *Lactobacillus johnsonii* MT4 reduced the metabolic activity of *C. albicans* biofilms in a dose–response pattern (Supplementary Figure 5). The ability of *C. albicans* to form biofilms was significantly hindered by the MT4 strain (Figure 7C), decreasing the fungal biofilm biovolumes (Figure 7E), thickness (Figures 7A,C,F, *side view*), and biomass as assessed by qPCR (Figure 7G). *L. johnsonii* ATCC 33200 displayed similar antibiofilm activity to strain MT4 (Figures 7D–G). In addition to hindering the formation of fungal biofilms, the yeast morphotype was present in the dual-species biofilms but was rarely observed in the single-species fungal biofilms. The pH of biofilm media at the end of single- and dual-species cultures were 6.08 ± 0.18 and 6.65 ± 0.44, respectively, suggesting that the effect of *L. johnsonii* on biofilm growth is not due to media acidification by the bacteria under these growth conditions.

**Figure 8:**
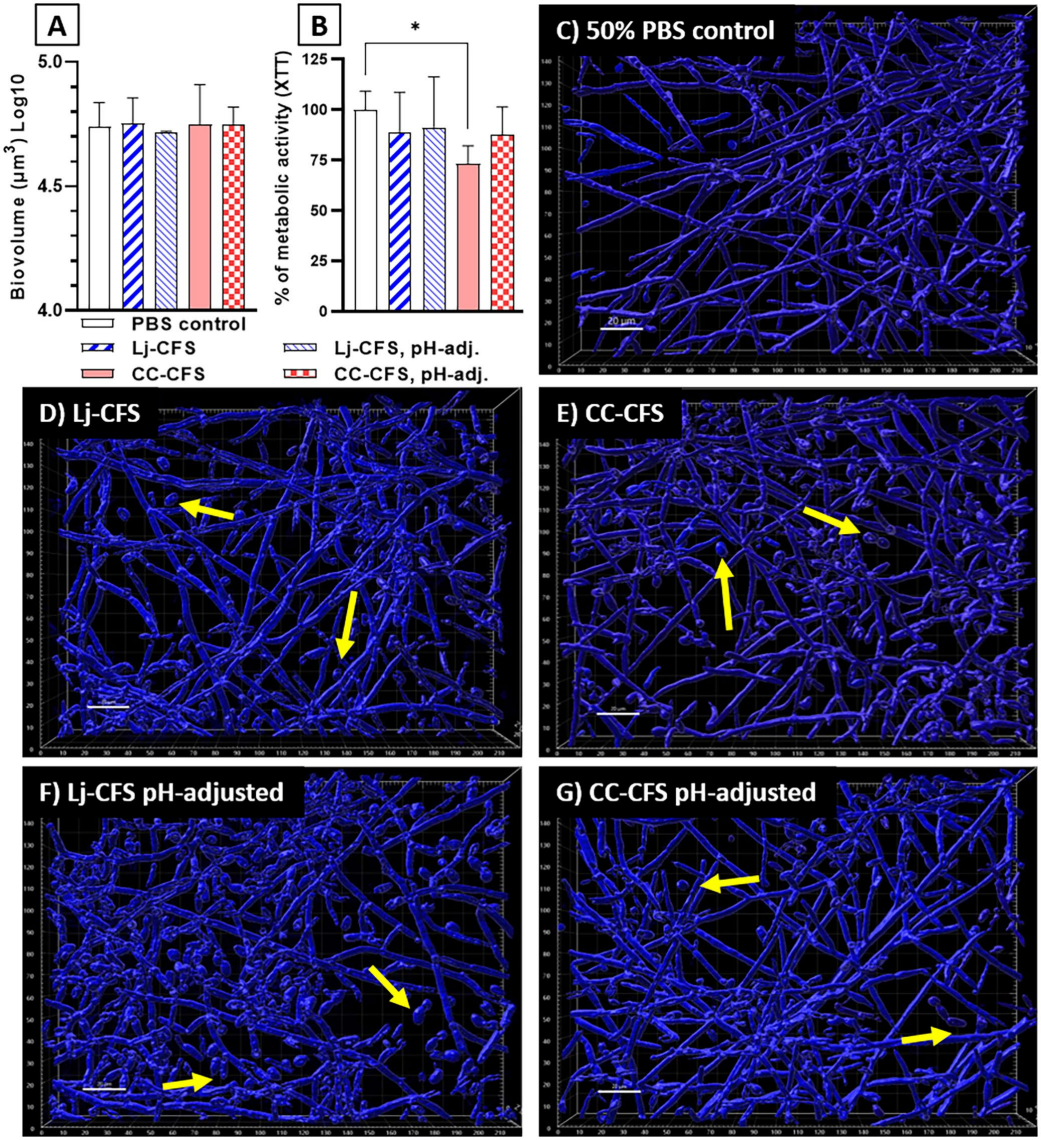
Cell-Free Supernatants reduce the ability of *C. albicans* for hyphal transition in biofilm growth. CFS were prepared from single *L. johnsonii* biofilms (Lj-CFS, panels **D**,**F**) or from biofilm cocultures with *C. albicans* (CC-CFS, panels **E**,**G**) in biofilm media and were added during biofilm growth. CSF were used with and without pH neutralization to discern the metabolites’ activity beyond acidification. CFS did not reduce the fungal biofilm biovolumes (panel **A**) but reduced the average metabolic activity of the biofilms **(B)**. Additionally, all CFS supplements notably increased the yeast morphotype numbers in biofilms. **(C–G)**. Yellow arrows indicate yeast cells. White bar = 20 μ. One-Way ANOVA, Dunnett posttest. *p ≤ 0.05. 100% of XTT metabolic activity (panel B) corresponds to OD_490_ = 0.48 ± 0.05 SD of biofilms in 50% PBS.

To examine the role of physical contact between the two organisms in *Candida* biofilm growth inhibition, we used transwell inserts during biofilm growth, allowing passage of soluble metabolites from lactobacilli seeded on the upper transwell compartment. Physical separation of microorganisms with transwell inserts did not significantly impact the reduction in *C. albicans* biofilm biomass, showing that physical contact between *L. johnsonii* and *C. albicans* is not required for inhibiting fungal biofilm growth (Supplementary Figure 6). Instead, these data suggested that either nutrient competition during coculture or secreted *L. johnsonii* metabolites are responsible for this effect. These results agree with studies using a similar design that showed other *Lactobacillus* species having biofilm inhibitory activity against *C. albicans* (Poupet et al., 2019) and *Streptococcus mutans* (Wu et al., 2015) in a contact-independent way. To further explore the impact of secreted metabolites, we tested the CFS prepared from biofilm cultures on fungal biofilm growth. CFS did not reduce the fungal biofilm biovolumes compared to the 50% PBS control (Figure 8A), but caused a small but statistically significant reduction in the metabolic activity of the fungal biofilms (Figure 8B). Importantly, CSF from *L. Johnsonii* alone or from coculture with *C. albicans* hindered its ability to transition into hyphae (Figures 8C–G). Surprisingly the yeast morphotype was the most abundant in all CFS-treated biofilms, regardless of whether the pH of the CSF was adjusted before adding to the culture media or not (Figures 8D–G). These results, taken together, suggest that *Lactobacillus* metabolites, accumulating over time during growth in biofilm medium, can negatively impact fungal metabolic activity and hinder the dimorphic transition in a pH-independent manner.

**Figure 9:**
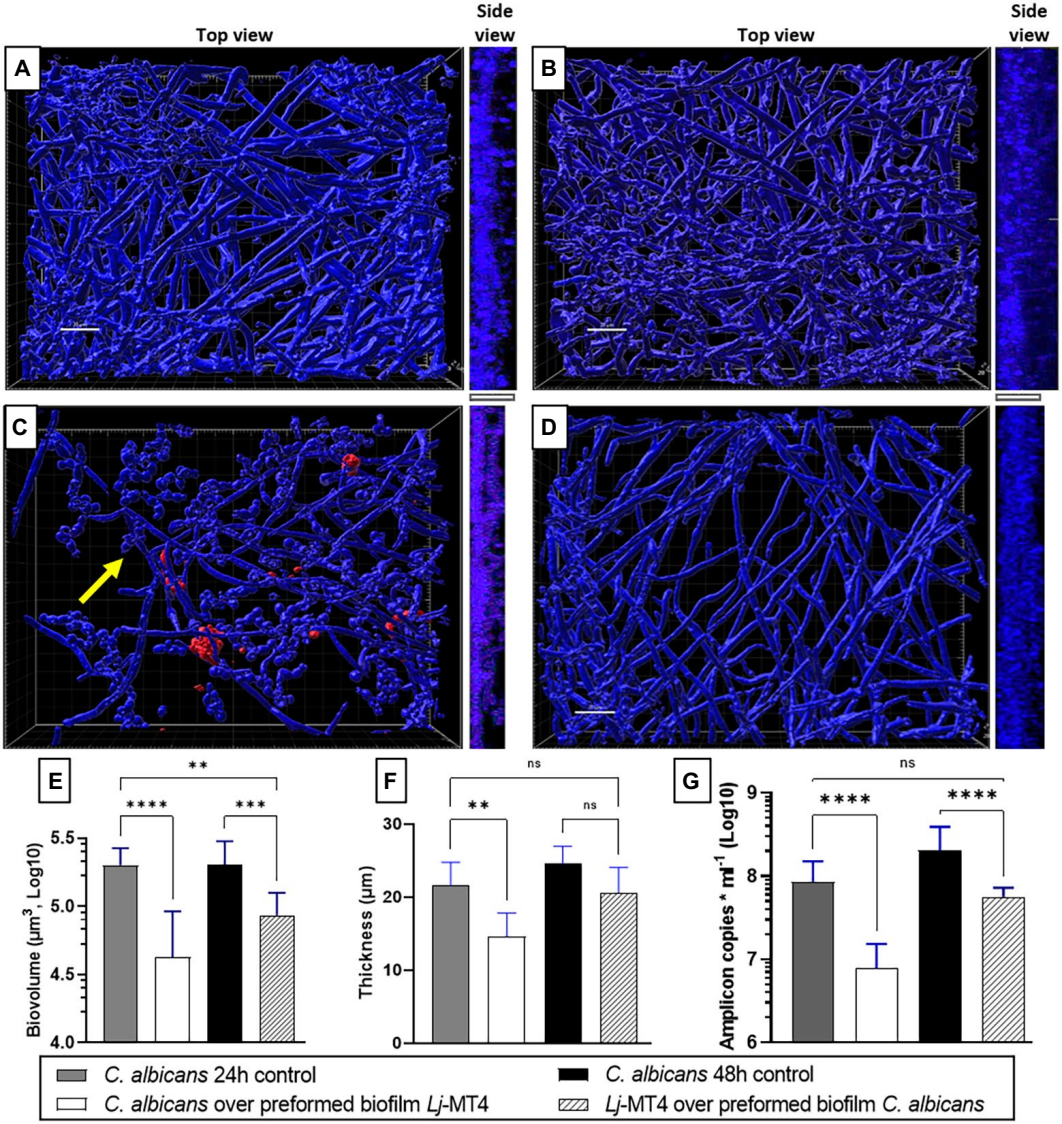
*Lactobacillus johnsonii* displays exclusion and displacement effects against *C. albicans* biofilms. **(A)** 24 h-old and **(B)** 48 h-old single *C. albicans* biofilms; **(C)** Addition of *C. albicans* on preformed *L. johnsonii* biofilm; **(D)** Addition of *L. johnsonii* over preformed *C. albicans* biofilm; Fungal biofilm **(E)** biovolumes, **(F)** thickness, and **(G)** biomass are significantly reduced in the presence of lactobacilli. Yellow arrows indicate the presence of yeast cells. White bar = 20 nm. One-Way ANOVA, Dunnett posttest. * *p* ≤ 0.05, ** *p* ≤ 0.01, ***p ≤ 0.005, **** *p* ≤ 0.0001.

Based on these observations, we next hypothesized that 24 h preformed biofilms of *L. johnsonii* will reduce the ability of *C. albicans* to form biofilms (Figures 9A,C,E–G). Indeed there was a significant reduction in biofilm growth as confirmed with biovolume (Figure 9E), thickness (Figures 9A,C,F, *side view*), and biomass (Figure 9G) estimates. As expected, a large number of cells remained in the yeast morphotype, suggesting that the *Lactobacillus* preformed biofilm reduced the dimorphic transition into hyphae, which is an essential stage in biofilm growth on mucosal surfaces (Chandra et al., 2001). Along the same lines, when lactobacilli were added to the 24-h *Candida* preformed biofilms, further growth of *C. albicans* biofilms was prevented (Figure 9D) as seen in comparison to 48 h control biofilms (Figures 9A,B). Thickness and biomass estimates in this setting were similar to the initial 24 h biofilm control, suggesting arrested biofilm growth. However, fungal biofilm biovolumes (Figure 9E) were significantly lower than both the 24 and 48 h control biofilms, possibly due to the fact that lactobacilli-treated biofilms were not uniformly distributed on the surface (Figure 9D). The ability of *L. johnsonii* to alter the fungal biofilm structure and prevent its further growth indicates an antagonistic relationship between these species.

## Discussion

In this work, we showed that, like other *Lactobacillus* species (Kang et al., 2018; Jang et al., 2019; Scillato et al., 2021), *L. johnsonii* displays anticandidal properties, reducing *C. albicans* growth and ability to transition into hyphae and establish biofilms on abiotic surfaces. We found that the MT4 strain shows strong dose-dependent anticandidal activity, particularly in the biofilm growth phase. The anticandidal activity of *L. johnsonii* depends on several factors such as viability, cell density, nutrient availability, production of metabolites, and partly on acidification, which are all influenced by growth conditions. Genomic analysis of this strain revealed the presence of several genes that encode metabolites with anticandidal properties, which may explain our results. These metabolites are analogous to other anticandidal compounds isolated and characterized from different bacterial species (i.e., *Bacillus subtilis* and other members of the genus *Lactobacillus*). For example, Bacillomycin D (from *B. subtilis*) is a fungicidal lipopeptide bacteriocin from the iturin group, which targets the cell membrane, creating ion-conducting pores due to the formation of lipopeptide–sterol complexes (Olfa et al., 2015). Additionally, Bacillomycin D-like peptides inhibit β-1,3-glucan synthesis, a major component of the fungal cell wall (Hajare et al., 2016). Surfactin, produced by different *Lactobacillus* species, is a cyclo-lipopeptide biosurfactant that reduces substrate adhesion of *C. albicans*, decreasing its ability to form biofilms (Nelson et al., 2020). The major secreted protein Msp1 is a hydrolase that cleaves chitin, one of the main biopolymers in the fungal cell wall. Recently, this hydrolase was implicated in inhibiting *C. albicans* morphogenesis into hyphae (Allonsius et al., 2019) and reducing the virulence of *C. glabrata* in a mouse model (Charlet et al., 2020). It is possible that any or all of these metabolites are involved in the anticandidal activities we observed *in vitro*. In addition to these metabolites, data from other lactobacilli (Bergsson et al., 2001; Schaefer et al., 2010; Gomaa, 2013; Charlet et al., 2020) suggest that *L. johnsonii* may encode novel antimicrobial products that require further characterization.

The role of lactate/lactic acid and media acidification on *C. albicans* has been widely discussed in the literature, sometimes with opposite conclusions. Köhler et al. showed that *C. albicans* growth was reduced in lactic acid-supplemented MRS; however, when the supplemented MRS was neutralized, *Candida* resumed its expected growth, suggesting that acidification and not lactate is the leading cause of growth inhibition (Köhler et al., 2012). In contrast, Lourenço et al. showed that *C. albicans* growth is not significantly reduced in lactic acid-supplemented minimal media under acidic conditions (Lourenço et al., 2019), implying that acidification is not relevant under the tested conditions. Our results show that the impact of acidification on *C. albicans* growth inhibition depends on the culture media, as acidification played a key role on MRS broth, but not in BHI or biofilm medium, which did not acidify significantly during co-culture. Also, the D−/L-lactate ratio was different across the growth media, with D-lactate the enantiomer primarily produced in MRS and the L enantiomer in BHI and biofilm medium. *Candida albicans* metabolizes L-lactate produced by the microbiota and the host, but cannot process D-lactate produced only by the microbiota. The ability of *C. albicans* to metabolize L-lactate produced in BHI and biofilm medium and neutralize the pH may be associated with the higher pH we observed in *Candida*-*Lactobacillus* co-cultures in these media compared to single cultures or cocultures in MRS (Danhof et al., 2016). Beyond its impact on fungal growth, lactic acid/lactate influence fungal physiology and morphology. Exposure for 76 h or longer to lactic acid, and other weak organic acids, turns the yeast cells into a “starvation-like” state, with slow growth rates and RNA-associated metabolism (Cottier et al., 2015). Additionally, lactate reduces the biosynthesis of ergosterol and induces incorrect localization of the transporter Cdr1, reducing the efflux of fluconazole in *C. albicans* cells (Suchodolski et al., 2021), leading to improving the efficacy of azoles, in combined treatments, against *C. albicans* (Alves et al., 2017; Lourenço et al., 2019). Disruption of ergosterol synthesis may interfere with fungal susceptibility to Bacillomycin D, which forms complexes with ergosterol, causing pores on the cell membrane. On the other hand, lactate also contributes to masking *C. albicans* β-glucans (Ene et al., 2013), allowing the fungi to elude the immune system. Yet, β-glucan masking is triggered by L-lactate but not by its D isomer (Ballou et al., 2016). The enantiomer produced by lactobacilli colonizing mucosal sites *in vivo* is unknown, but our results in the serum-supplemented biofilm media suggest that the L-enantiomer may be more physiologically relevant and thus likely to play a significant role in fungal recognition by innate immune cells and the overall host-microbiota-Candida interactions.

*Lactobacillus johnsonii* MT4 displayed an auto-aggregation phenotype. While not all strains of *L. johnsonii* display this phenotype (Jankovic et al., 2003), aggregation is a relevant trait in several ecological niches, including within the host mucosal sites, as it promotes the interaction between microbial cells. Similarly, as suggested for other *L. johnsonii* strains (Gil et al., 2010), MT4 can co-aggregate with *C. albicans*. In the context of the oral cavity, co-aggregation is a physical interaction mechanism that positively influences colonization and biofilm formation (Kolenbrander, 2000).

We observed that preformed biofilms of lactobacilli significantly inhibited fungal biofilms and the yeast’s ability to shift to hyphae. These properties of the mouse MT4 strain may explain the dominant yeast phenotype of *C. albicans* on the oral mucosa of mice receiving a sucrose-rich diet which promotes the growth of lactobacilli (Souza et al., 2020; Bertolini et al., 2021). In addition to preventing *Candida* from forming biofilms, *L. johnsonii* MT4 disrupted preformed fungal biofilms, implying that these bacteria may also curtail further growth of biofilms in colonized surfaces. Quorum sensing (QS) regulates *C. albicans* yeast-to-hyphal transition and biofilm formation (Kruppa, 2009). Diverse QS molecules (i.e., farnesol and fatty acids) can prevent yeast from shifting into hyphae (Lee et al., 2021). Lactobacilli can inhibit QS-induced biofilms in bacteria (Aman et al., 2021), but their impact on *C. albicans* QS responses requires further investigation.

In conclusion, we showed that *L. johnsonii* has an antagonistic relationship with *C. albicans* during planktonic and biofilm growth *in vitro*. Environmental variables, such as the type and amount of nutrients, influence *L. johnsonii* MT4 metabolism, and anticandidal activity. Genomic analysis revealed that beyond acidification and lactate, other soluble metabolites may be responsible for the anticandidal activity and are the focus of current and future investigations. Our findings suggest that this species displays promising probiotic properties to prevent or treat mucosal candidiasis.

## Data Availability Statement

The raw FASTQ sequencing data are available at the NCBI Sequence Read Archive under accession SRR17309641. The assembled MT4 genome sequence was submitted to the NCBI Genome database with accession JAJQJG000000000. The MT4 BioSample SAMN23838460 and other associated data listed previously are available under BioProject PRJNA787656.

## Author Contributions

RV-M: experimental work, analysis, and writing. AT: resources and experimental work. JR: genomic analysis and repositories deposition. TS: resources. YZ: supervision and genomic analysis. AD-B: project administration, funding acquisition, supervision, and writing. All authors contributed to the article and approved the submitted version.

## Funding

This study was funded by NIH/NIDCR grant RO1 DE013986 and NIH/NIGMS grant RO1 GM127909.

## Conflict of Interest

The authors declare that the research was conducted in the absence of any commercial or financial relationships that could be construed as a potential conflict of interest.

## Publisher’s Note

All claims expressed in this article are solely those of the authors and do not necessarily represent those of their affiliated organizations, or those of the publisher, the editors and the reviewers. Any product that may be evaluated in this article, or claim that may be made by its manufacturer, is not guaranteed or endorsed by the publisher.

## Footnotes

^1^ http://www.atgc-montpellier.fr/sms/
