# Supplementary material for: Insights From the Lactobacillus johnsonii Genome Suggest the Production of Metabolites With Antibiofilm Activity Against the Pathobiont Candida albicans
Source: Front Microbiol. 2022 Mar 7;13:853762. doi: 10.3389/fmicb.2022.853762 (PMC8940163; doi:10.3389/fmicb.2022.853762)
Supplement: Supplementary file 1 [file Data_Sheet_1.docx]

Supplementary Material

Insights from the *Lactobacillus johnsonii* genome suggests the production of metabolites with antibiofilm activity against the pathobiont *Candida albicans*

# Supplementary Figures and Tables

## Supplementary Figures

**Supplementary Figure S1**


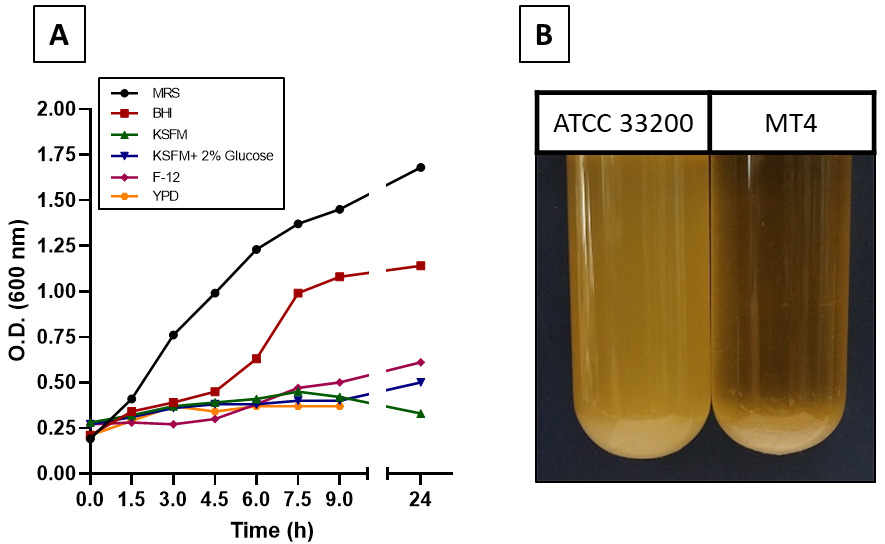


**Supplementary Figure S1**. Phenotypical characterization. The growth of *L. johnsonii* MT4 –in different culture media- and its auto-aggregation phenotype were characterized. A) Growth curve of *L. johnsonii* MT4 growing in different culture media, at 37 ^o^C, aerobically with 5 % CO_2_, for 24 h. The Optical Density at λ=600 nm (OD_600_) was measured every 90 min. *L. johnsonii* displayed the best growth rate on MRS broth, followed by BHI broth. B) Overnight cultures on MRS growth show differences in the auto-aggregation *L. johnsonii* MT4 and ATCC 33200 strains. Strain MT4 displays an auto-aggregation phenotype; in contrast, the ATCC 33200 strain is non-aggregating.

**Supplementary Figure S2**


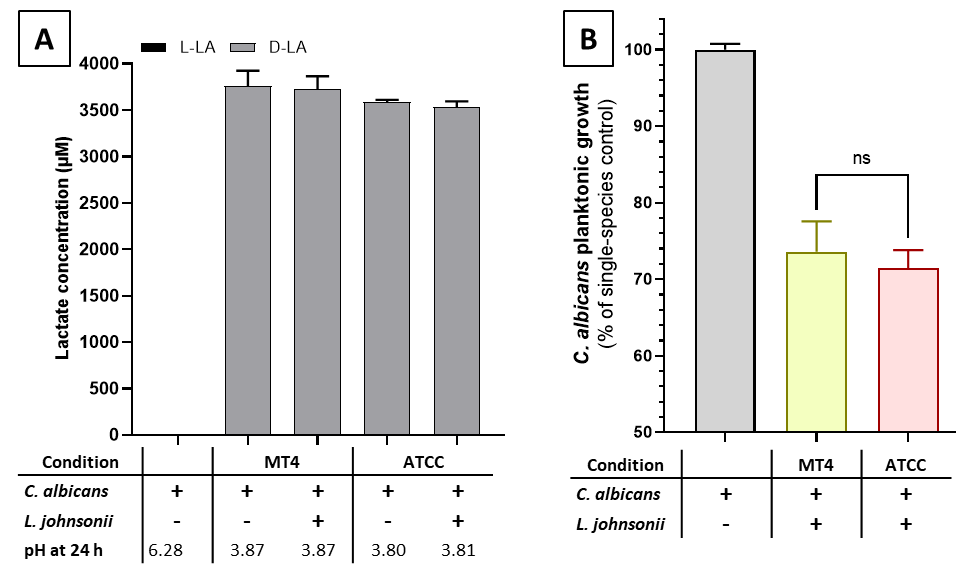


**Supplementary Figure S2**. DL-lactate production and anticandidal activity of *L. johnsonii* ATCC 33200 compared to the *L. johnsonii* MT4 isolate. ATCC 33200 strain displays similar anticandidal activity and DL-lactate production as the MT4 isolate in MRS broth. One-Way ANOVA, Dunnett posttest. 100% growth (panel B) corresponds to 8.16 ±0.41 SD yeast cells ml^-1^ (average, log10 values) of single-species culture in MRS broth.

**Supplementary Figure S3**


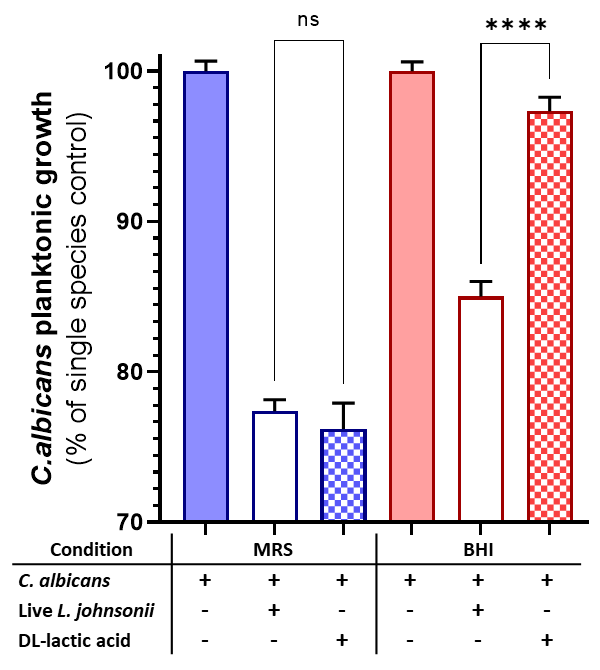


**Supplementary Figure S3**. Effect of lactic-acid acidified media on *C. albicans* growth. MRS or BHI media were supplemented with sufficient lactic acid to lower the pH to levels comparable with a 24h culture of *L. johnsonii* (~3.9 for MRS and ~5.5 for BHI). Lactic acid acidification caused a similar *C. albicans* growth inhibition in MRS media compared to co-culture with live lactobacilli. In contrast, growth inhibition in lactic-acid supplemented BHI was significantly lower than that induced by live bacteria. One-Way ANOVA, Dunnett posttest, ****= p≤0.0001. 100% growth corresponds to 8.16 ±0.41 SD and 7.47 ±0.08 SD yeast cells ml^-1^ (average, log10 values) in MRS and BHI broth, respectively.

**Supplementary Figure S4**


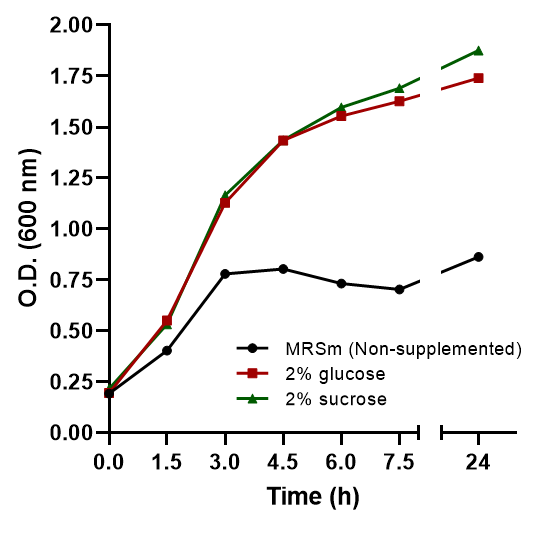


**Supplementary Figure S4**. Carbohydrate availability influenced *Lactobacillus* fitness. In carbohydrate-free MRSm, there was a significantly reduced *L. johnsonii* MT4 growth rate. Supplementing the media with either 2% dextrose or 2% sucrose fully restored the bacterial growth.

**Supplementary Figure S5**


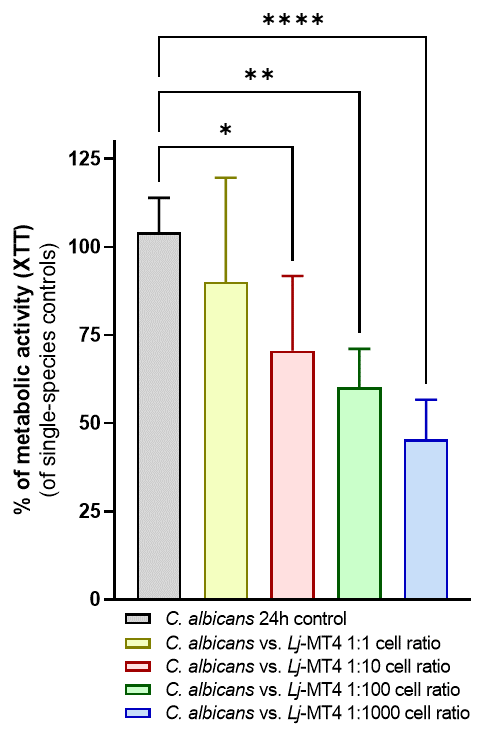


**Supplementary Figure S5**. *L. johnsonii* reduces the metabolic activity of the *C. albicans* biofilms. The fungal metabolic activity was decreased in the presence of *L. johnsonii* MT4 in a dose-response pattern during the biofilm formation stage. One-Way ANOVA, Dunnett posttest. *= p≤0.05, **= p≤0.01, ****= p≤0.0001. 100% of XTT metabolic activity corresponds to OD_490_= 0.74 ±0.04 SD, in 24 h single-species biofilms.

**Supplementary Figure S6**


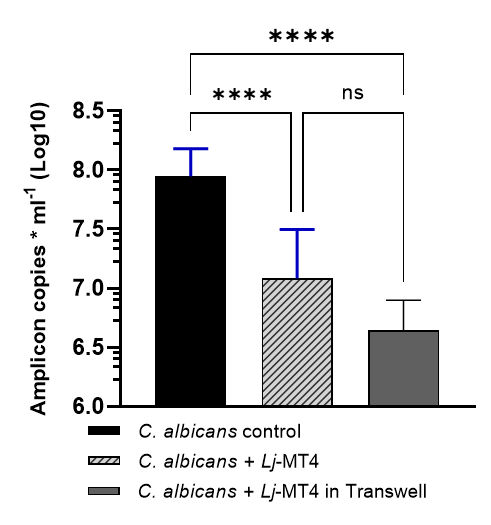


**Supplementary Figure S6**. *L. johnsonii* MT4, seeded inside transwell inserts, showed an antibiofilm activity against *C. albicans* similar to the dual-cultures grown together, suggesting that physical contact is not required. One-Way ANOVA, Dunnett posttest. ****= p≤0.0001.

## Supplementary Tables

**Supplementary Table S1**

| **Sup. Table S1.** Kraken2 taxonomic classification results confirm the identity of MT4 strain as *L. johnsonii* | | | | | |
| --- | --- | --- | --- | --- | --- |
| **% ^1^** | # of fragments | | Rank ^4^ | TaxID ^5^ | Scientific name ^6^ |
|  | Covered^2^ | Assigned^3^ |  |  |  |
| **100.00** | 68 | 0 | R | 1 | root |
| **100.00** | 68 | 0 | R1 | 131567 | cellular organisms |
| **100.00** | 68 | 0 | D | 2 | Bacteria |
| **100.00** | 68 | 0 | D1 | 1783272 | Terrabacteria group |
| **100.00** | 68 | 0 | P | 1239 | Firmicutes |
| **100.00** | 68 | 0 | C | 91061 | Bacilli |
| **100.00** | 68 | 0 | O | 186826 | Lactobacillales |
| **100.00** | 68 | 0 | F | 33958 | Lactobacillaceae |
| **100.00** | 68 | 0 | G | 1578 | Lactobacillus |
| **98.53** | 67 | 40 | S | 33959 | *Lactobacillus johnsonii* |
| **14.71** | 10 | 10 | S1 | 1408186 | *Lactobacillus johnsonii N6.2* |
| **13.24** | 9 | 9 | S1 | 257314 | *Lactobacillus johnsonii NCC 533* |
| **7.35** | 5 | 5 | S1 | 909954 | *Lactobacillus johnsonii DPC 6026* |
| **4.41** | 3 | 3 | S1 | 633699 | *Lactobacillus johnsonii FI9785* |
| **1.47** | 1 | 1 | S | 47770 | *Lactobacillus crispatus* |

^1^ Percentage of fragments covered by the clade rooted at this taxon

^2^ Number of fragments covered by the clade rooted at this taxon

^3^ Number of fragments assigned directly to this taxon

^4^ Rank: (U) Unclassified, (R) Root, (D) Domain, (K) Kingdom, (P) Phylum, (C) Class, (O) Order,
(F) Family, (G) Genus, or (S) Species.

^5^ NCBI taxonomic ID number

^6^ Indented scientific name

**Supplementary Table S2**

| **Supplementary Table S2**. BLAST analysis for the *L. johnsonii* strain MT4 metabolites associated with the anticandidal activity. | | | | |
| --- | --- | --- | --- | --- |
| **Metabolite** | **Coverage** | **Identity** | **Score (Max/Total)** | **E-value** |
| Bacillomycin D | 36 % (contig 11) | 30.28 % | 187/684 | 1e^-47^ |
| Surfactin | 40 % (contig 11) | 28.91 % | 174/491 | 7e^-44^ |
| Glucanase | 89 % (contig 2) | 31.21 % | 144/144 | 6e^-39^ |
| Msp1/p75 | 24 % (contig 3)  13 % (contig 7) | 49.17 %  30.88 % | 126/126  32.7/32.7 | 6e^-32^  3.6e^-2^ |
